# 9-Phenanthrol, a TRPM4 Inhibitor, Protects Isolated Rat Hearts from Ischemia–Reperfusion Injury

**DOI:** 10.1371/journal.pone.0070587

**Published:** 2013-07-25

**Authors:** Jing Wang, Ken Takahashi, Hulin Piao, Peng Qu, Keiji Naruse

**Affiliations:** 1 Department of Cardiology, Graduate School, Dalian Medical University, Dalian, China; 2 Department of Cardiovascular Physiology, Graduate School of Medicine, Dentistry and Pharmaceutical Sciences, Okayama University, Okayama, Japan; 3 Department of Cardiovascular Surgery, The Second Affiliated Hospital of Jilin University, Changchun, China; 4 Department of Cardiology, The Second Affiliated Hospital of Dalian Medical University, Dalian, China; University of Otago, New Zealand

## Abstract

Despite efforts to elucidate its pathophysiology, ischemia–reperfusion injury lacks an effective preventative intervention. Because transient receptor potential cation channel subfamily M member 4 (TRPM4) is functionally expressed by many cell types in the cardiovascular system and is involved in the pathogenesis of various cardiovascular diseases, we decided to assess its suitability as a target of therapy. Thus, the aim of this study was to examine the possible cardioprotective effect of 9-phenanthrol, a specific inhibitor of TRPM4. Isolated Langendorff-perfused rat hearts were pretreated with Krebs–Henseleit (K–H) solution (control), 9-phenanthrol, or 5-hydroxydecanoate (5-HD, a blocker of the ATP-sensitive potassium channel) and then subjected to global ischemia followed by reperfusion with the K–H solution. To evaluate the extent of heart damage, lactate dehydrogenase (LDH) activity in the effluent solution was measured, and the size of infarcted area of the heart was measured by 2,3,5-triphenyltetrazolium chloride staining. In controls, cardiac contractility decreased, and LDH activity and the infarcted area size increased. In contrast, in hearts pretreated with 9-phenanthrol, contractile function recovered dramatically, and the infarcted area size significantly decreased. The cardioprotective effects of 9-phenanthrol was not completely blocked by 5-HD. These findings show that 9-phenanthrol exerts a cardioprotective effect against ischemia in the isolated rat heart and suggest that its mechanism of action is largely independent of ATP-sensitive potassium channels.

## Introduction

Despite significant advances in therapeutic techniques, ischemic heart disease remains the leading cause of mortality and heart failure in most countries [1]. Although early reperfusion can salvage the myocardium after ischemia, reperfusion induces myocardial injury called “reperfusion injury,” which attenuates the benefits of primary percutaneous coronary intervention and thrombolytic therapy [2]. Thus, the development of more effective drugs or interventions to protect the myocardium from reperfusion injury is required to provide greater clinical benefits for patients with ischemic heart disease [3].

Ischemic preconditioning, defined as a resistance to infarction induced by ischemia/reperfusion (I/R) [4], significantly reduces infarct size, arrhythmia, and contractile dysfunction. Numerous studies have shown that ischemic preconditioning can be mimicked by techniques such as pharmacological stimulation [5], heat-shock preconditioning [6], and mechanical stretching of the heart [7].

The transient receptor potential cation channel subfamily M member 4 (TRPM4) is a potential target for this approach. TRPM4 is activated following receptor mediated calcium mobilization and represents a regulatory mechanism that controls the magnitude of calcium influx by modulating the membrane potential and the driving force for calcium entry through other calcium-permeable pathways [8]. This channel is widely expressed and is particularly abundant in the heart tissue. Several studies have demonstrated that mutations in the human gene encoding TRPM4 are associated with cardiac conduction block [9], [10]. The most specific inhibitor of TRPM4 channels currently available is 9-phenanthrol [11], [12], which abolishes arrhythmias induced by hypoxia and reoxygenation in the mouse ventricle [13]. Despite these reports, the physiological and pathological role of TRPM4 in heart function is poorly understood.

The primary aim of the present study was to assess the cardioprotective effect of 9-phenanthrol on isolated rat heart and to explore the possible cardioprotective mechanisms. To the best of our knowledge, this is the first report demonstrating cardioprotective effects of 9-phenanthrol.

## Materials and Methods

### Animals

Male Sprague–Dawley rats aged 13–15 weeks were used in this study. The Animal Care and Use Committee of Okayama University approved our protocol for conducting animal experiments (Permit Number: OKU-2012351 and OKU-2012522). All surgery was performed under sodium pentobarbital anesthesia, and every effort was made to minimize suffering.

### Langendorff Heart Preparation

Rats were anesthetized by intraperitoneal injection of pentobarbital sodium (60 mg/kg body weight). Hearts were rapidly excised, connected immediately to an aortic cannula, and subjected to retrograde perfusion at a constant pressure (70–80 mmHg) in the Langendorff apparatus with the K–H buffer (118.5 mM, NaCl; 4.7 mM, KCl; 2.5 mM, CaCl_2_·2H_2_O; 1.2 mM, MgSO_4_; 11 mM, glucose; and 25 mM NaHCO_3_). The buffer solution was saturated with a mixture of 95% O_2_/5% CO_2_ at 37°C [14]. To measure the left ventricular pressure (LVP), a small balloon tip catheter was inserted into the left ventricle through the left auricular appendage. The isolated heart was placed in a water jacket and maintained at 37°C at all times. The balloon was inflated until the end diastolic pressure reached 6–10 mmHg. Special care was taken to maintain the diastolic pressure at<10 mmHg to avoid stretch-induced preconditioning [7]. Pacing electrodes were fixed to the right auricular appendage to induce sufficient myocyte damage during ischemia. Global ischemia was induced by stopping the pump and performing pacing at 5.0 Hz (voltage, 5.0 V; duration, 2.0 ms) using an electrical stimulator (SEN-3301, Nihon Kohden, Tokyo, Japan) equipped with an isolator (SS-102J, Nihon Kohden). Pacing was applied only during the ischemic procedure.

### Experimental Protocol

**Figure 1:**
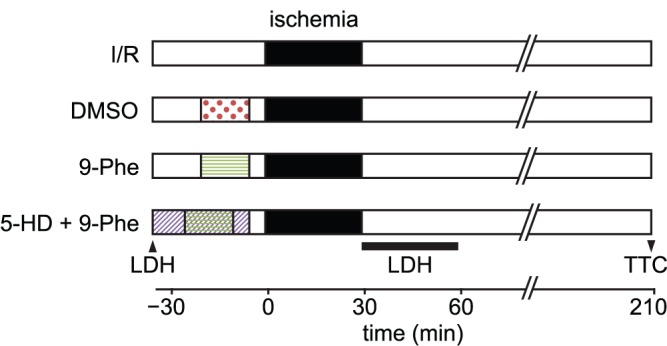
Experimental protocol. All experimental groups were first perfused for 20 min to allow the isolated hearts to stabilize. The hearts were then divided into groups as follows: ischemia–reperfusion (I/R), DMSO-treated, 9-Phe-treated, and 5-HD+9-Phe-treated. LDH measurement was carried out before drug application and after ischemia. Finally, the heart was used for TTC analysis.

After waiting for at least 20 min for heart activity to stabilize, hearts were perfused for 30 min (pre-ischemia), 30 min of global ischemia, and 180 min of reperfusion. The hearts were divided into the following four groups as shown in Figure 1: the I/R control group (n = 6) was subjected to 30 min of global ischemia followed by 180 min of reperfusion. The groups treated with dimethyl sulfoxide (DMSO, n = 7) or 20 µM 9-phenanthrol (9-Phe, n = 8) were subjected to perfusion for 15 min with the K–H buffer containing 0.0067% DMSO or 20 µM 9-phenanthrol (dissolved in DMSO), respectively, followed by washing out of the drug by perfusion with the K–H buffer for 5 min before inducing ischemia. Another group was sequentially treated with 9-phenanthrol and 5-HD (5-HD+9-Phe, n = 6) as follows: 10 min of 5-HD, 15 min of 9-phenanthrol and 5-HD, and 5 min of 5-HD, followed by application of the K–H buffer for 5 min (Figure 1). Treatment with 5-HD is known to inhibit the cardioprotective effect of ischemic preconditioning by blocking K_ATP_ channels [15], [16].

### Measurement of Contracting Function

Myocardial contractility was assessed by measuring left ventricular developed pressure (LVDP), which was calculated by subtracting the left ventricular end diastolic pressure values from LVP peak values. Data are expressed as a percentage of their respective values before drug administration.

### Epicardial Electrocardiogram (ECG)

The epicardial ECG was continuously recorded from electrodes placed on the aorta and the ventricular apex. Electrical signals were amplified by bioelectric amplifiers (AB-601G, Nihon Kohden) with a time constant of 0.1 s, and recorded after digitization through a recording system (NI cDAQ-9174, National Instruments, Austin, TX, USA) at a sampling frequency of 1 kHz.

### Measurement of Lactate Dehydrogenase (LDH) Activity

The release of LDH, which indicates tissue damage, was measured in samples collected from coronary effluents before drug administration and the first 30 min of reperfusion for all groups and assayed using an LDH Cytotoxicity Assay Kit (Cayman Chemical Company, Ann Arbor, MI, USA). The value is expressed in units per gram of heart wet-weight per liter (U/g/L).

### Measurement of Infarct Size

Infarct size was measured by staining hearts with 2,3,5-triphenyltetrazolium chloride (TTC) [17]. After Langendorff perfusion, the hearts were frozen by placing them in a freezer (−30°C) for 1–2 h. The hearts were then sliced into 2–3 mm thick sections perpendicularly to the long axis and incubated in 1% of 2,3,5-triphenyltetrazolium chloride in phosphate-buffered saline (PBS) for 20 min in a 37°C incubator followed by fixation for 10 min in 4% paraformaldehyde. Images of all slices were acquired using a FUJIFILM LAS-3000 system. Finally, the infarcted area was analyzed using Image-J software [18], and normalized infarct size (percentage) was derived by dividing the calculated total infarct size with the total heart volume. The size of the infarcted area was determined by assigning a fixed threshold value of brightness for all the images. Brightness was measured using a computer to avoid subjective evaluation by the experimenter.

### Detection of Apoptosis

Nuclear DNA fragmentation yielded by cleavage of genomic DNA during apoptosis was detected by terminal deoxynucleotidyl transferase dUTP nick end labeling (TUNEL) staining using a commercial kit (ApopTag Peroxidase In Situ Apoptosis Detection Kit, Merck Millipore, Bedford, MA, USA) according to the manufacturer’s instructions. Rat hearts were immersed in 4% paraformaldehyde phosphate buffer solution (Wako, Osaka, Japan) immediately after the Langendorff experiments and stored at 4°C (n = 6 for the I/R group and n = 6 for the 9-Phe-treated group). After 24 h, the hearts were immersed in 70% ethanol solution and stored at 4°C until the paraffin embedding procedure. Five-micrometer-thick sections of the rat heart in the transverse plane at the midpoint between the aorta and the ventricular apex were used. Samples were counterstained with DAPI and analyzed under fluorescence microscopy (LSM 780, Carl Zeiss, Germany). Fluorescein-stained TUNEL-positive areas were determined for whole areas of the paraffin section and were expressed as a percentage of the DAPI-positive area. Hematoxylin-eosin (HE) and Periodic Acid-Schiff (PAS) stainings were performed for the serial heart sections used in the TUNEL assay for histological analysis. Since Purkinje cells of the cardiac conduction system have rich glycogen particles, they can be visualized by PAS staining [19], [20].

### Statistical Analysis

All data are expressed as mean ± standard error of the mean (SEM) and were analyzed using Prism software (version 5.0, Graphpad Software, La Jolla, CA, USA). For analysis of LVDP and LDH activity, two-way analysis of variance (ANOVA) was performed followed by Bonferroni post hoc tests. For analysis of TTC staining, the Kruskal–Wallis test was performed followed by Dunn’s post hoc tests. For analysis of apoptosis rate, Student’s t-test was performed. *p*<0.05 was considered significant.

## Results

Protective effects of 9-phenanthrol against I/R in the isolated hearts were assessed by determining the recovery of heart function and the changes in normalized infracted size after I/R.

### Effect of 9-phenanthrol on Cardiac Function

**Figure 2:**
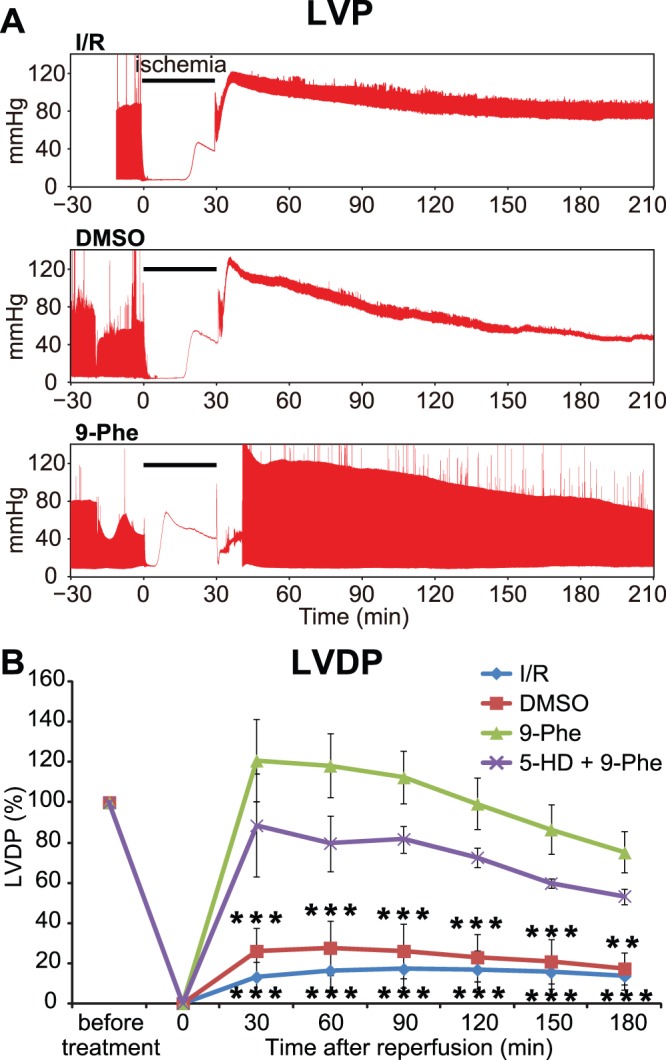
Measurement of cardiac contractile function. **A.** Representative recordings of left ventricular pressure (LVP). In the I/R and DMSO groups, the cardiac contraction decreased severely after the reperfusion. In contrast, LVDP recovered dramatically at the end of the reperfusion period in the 9-Phe-treated group. **B.** Contractile function of the heart indicated by the left ventricular developed pressure (LVDP). Impaired contractile function caused by I/R injury was significantly recovered by treatment with 9-Phe. Simultaneous application of the K_ATP_ channel-blocker, 5-HD, did not significantly change the cardioprotective effect of 9-Phe. Asterisks indicate a significant difference compared with the 9-Phe group as determined using Bonferroni post hoc tests.***p<0.001,**p<0.01.

**Figure 3:**
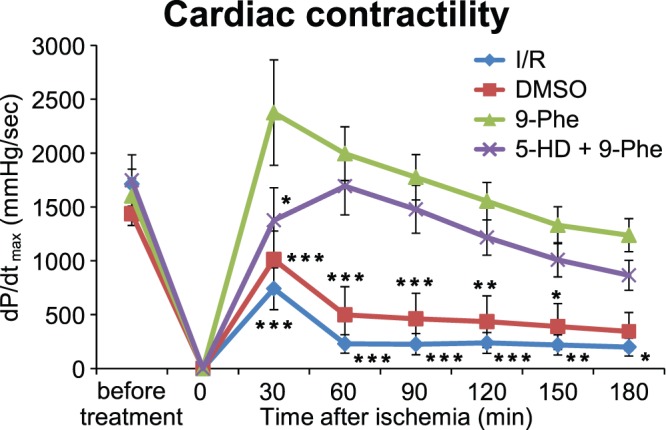
Analysis of heart contractility. Maximum value of the time derivative of LVP (dP/dt_max_) was used as an indicator for cardiac contractility. In the I/R and DMSO groups, decrease in dP/dt_max_ is evident. In contrast, impaired contractility caused by I/R injury was significantly recovered by treatment with 9-Phe. Asterisks indicate a significant difference compared with the 9-Phe group as determined using Bonferroni post hoc tests.***p<0.001,**p<0.01,*p<0.05.

Cardiac function was evaluated by measuring LVDP. Figure 2A shows the recordings of the change in the LVP during Langendorff perfusion. In contrast to the I/R group, both the contractile and diastolic functions recovered dramatically in the group pretreated with 9-phenanthrol. Figure 2B shows the LVDP change during Langendorff perfusion. After 30 min of global ischemia, LVDP in the I/R (n = 6) and DMSO (n = 6) groups recovered by only 13±6% and 17±8%, respectively, at the end of the 3 h period of reperfusion. In contrast, LVDP recovered by 75±10% in the 9-phenanthrol-treated group (n = 8), indicating that cardiac function was significantly protected from ischemic–reperfusion injury by treatment with 9-phenanthrol (I/R vs. 9-phenanthrol: *p*<0.001; DMSO vs. 9-phenanthrol: *p*<0.01). Furthermore, the protective effect was not significantly modified in the 5-HD group (n = 5). Cardiac contractility was further analyzed using the maximum value of the time derivative of LVP (dP/dt_max_, Figure 3). In contrast to the I/R group, the cardiac contractility recovered significantly in the group pretreated with 9-Phe. After 30 min of global ischemia, dP/dt_max_ in the I/R group (n = 6) recovered by only 199±82 mmHg/s at the end of the 3-h period of reperfusion. In contrast, dP/dt_max_ recovered by 1237±153 mmHg/s in the 9-Phe-treated group (n = 8), indicating that cardiac contractility was significantly protected from ischemic–reperfusion injury by treatment with 9-Phe (I/R vs. 9-Phe: *p*<0.05). dP/dt_max_ in the 5-HD group was 1376±302 mmHg/s at 30 min after ischemia–reperfusion, which was significantly lower than that in the 9-Phe-treated group (2375±490 mmHg/s, *p*<0.05).

**Figure 4:**
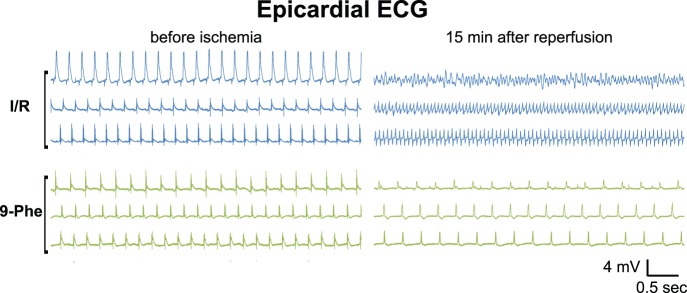
Representative epicardial ECGs from Langendorff excised hearts. Three representative recordings are shown for the control and 9-Phe-treated groups. While ventricular fibrillation or tachycardia was evident at 15 min after ischemia–reperfusion in the control group, regular rhythm was maintained in the 9-Phe-treated group.

**Figure 5:**
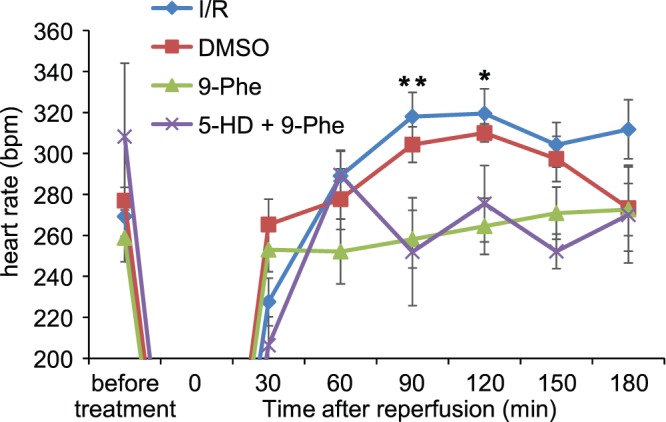
Measurement of heart rate. While heart rate increased after ischemia–reperfusion in the control group, that in 9-Phe-treated group was relatively stable. Simultaneous application of K_ATP_ channel inhibitor 5-HD did not affect the action of 9-Phe. Asterisks indicate a significant difference compared with the 9-Phe group as determined using Bonferroni post hoc tests.**p<0.01,*p<0.05.

To evaluate the effect of 9-phenanthrol on cardiac conduction after ischemic–reperfusion injury, epicardial ECG was recorded (Figure 4). In the I/R group, ventricular fibrillation or ventricular tachycardia was frequently observed at a time point of 15 min after reperfusion. In contrast, no abnormality was detected in the 9-Phe-treated group at the same time point. In addition, heart rate was stable in the 9-Phe-treated group compared to the I/R group (Figure 5). In this analysis, several data including invalid counts because of contractile abnormalities such as fibrillation, at any time point were excluded (n = 2 of 6 in the I/R, n = 3 of 6 in the DMSO, n = 1 of 8 in the 9-Phe, and n = 1 of 5 in the 5-HD groups). The heart rate in the I/R group at 90 min after reperfusion was increased to 318±12 bpm from the pre-ischemia value of 269±10 bpm. In contrast, the heart rate in the 9-Phe treated group was 258±14 bpm at 90 min after reperfusion (*p*<0.01 vs I/R group), which was the same as the pre-ischemia value of 259±12 bpm.

### Effect on LDH Release

**Figure 6:**
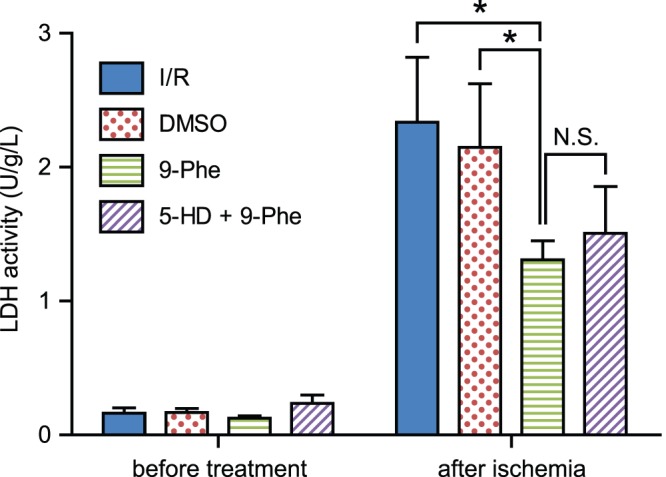
Determination of LDH activity to assess tissue damage. The LDH activity of the 9-Phe group was significantly lower than that of the I/R and DMSO groups after ischemia–reperfusion. Simultaneous application of K_ATP_ channel inhibitor 5-HD did not affect the action of 9-Phe.*p<0.05, N.S. *p*>0.05.

To measure the extent of cellular damage within the heart, the level of LDH activity of the effluent solution was measured (Figure 6). The level of LDH activity after ischemia–reperfusion injury was significantly higher in the I/R group (2.34±0.49 U/g/L; *p*<0.05; n = 6) and DMSO group (2.15±0.48 U/g/L; *p*<0.05; n = 7) than in the 9-Phe group (1.31±0.14 U/g/L; n = 8). The difference in LDH activities between 9-Phe and 5-HD (1.51±0.35; n = 6) was not significant (*p*>0.05).

### Infarct Size

**Figure 7:**
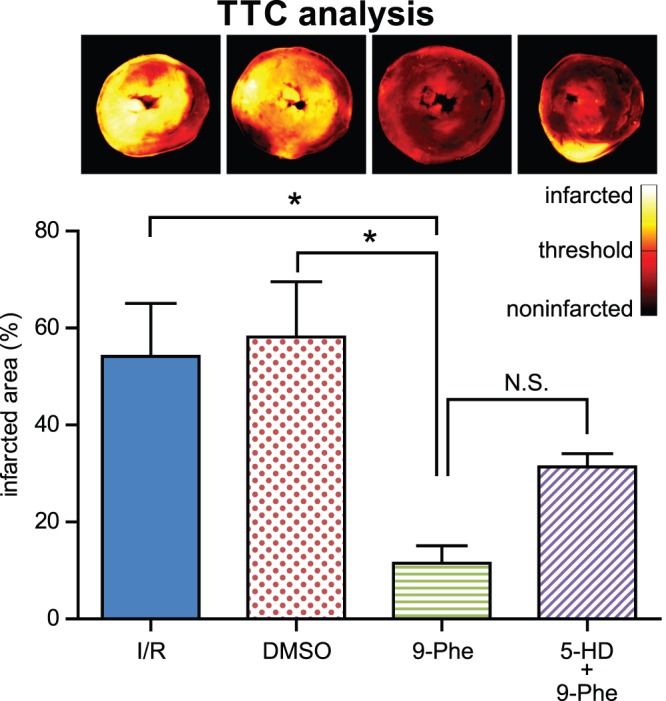
Analysis of infarction size. Images above the graph show the typical TTC-stained heart slices corresponding to the groups indicated below on the X-axis of the graph. The colored bar below the images indicates the brightness of the images. A fixed detection threshold for infarcted area is arbitrarily set and used throughout the analysis. The graph shows the percentage infarction in each group. The size of the infarcted area in the 9-Phe group was significantly smaller than that of the I/R and DMSO groups.*p<0.05, N.S. *p*>0.05.

Cellular damage in the heart was measured anatomically using TTC staining (Figure 7). The ischemic infarct size was 54±11% in the I/R group (n = 6), and the infarcted area was concentrated mostly in the left ventricle. The infarcted area was significantly smaller in the 9-Phe group (12±4%; n = 8) than in the I/R (*p*<0.05; n = 6) and DMSO (58±11%; *p*<0.05; n = 6) groups. Although the infarcted area in the 5-HD group (31±3%; n = 5) tended to be larger than that in the 9-Phe group, the difference was not significant (*p*>0.05).

### Cellular Apoptosis

**Figure 8:**
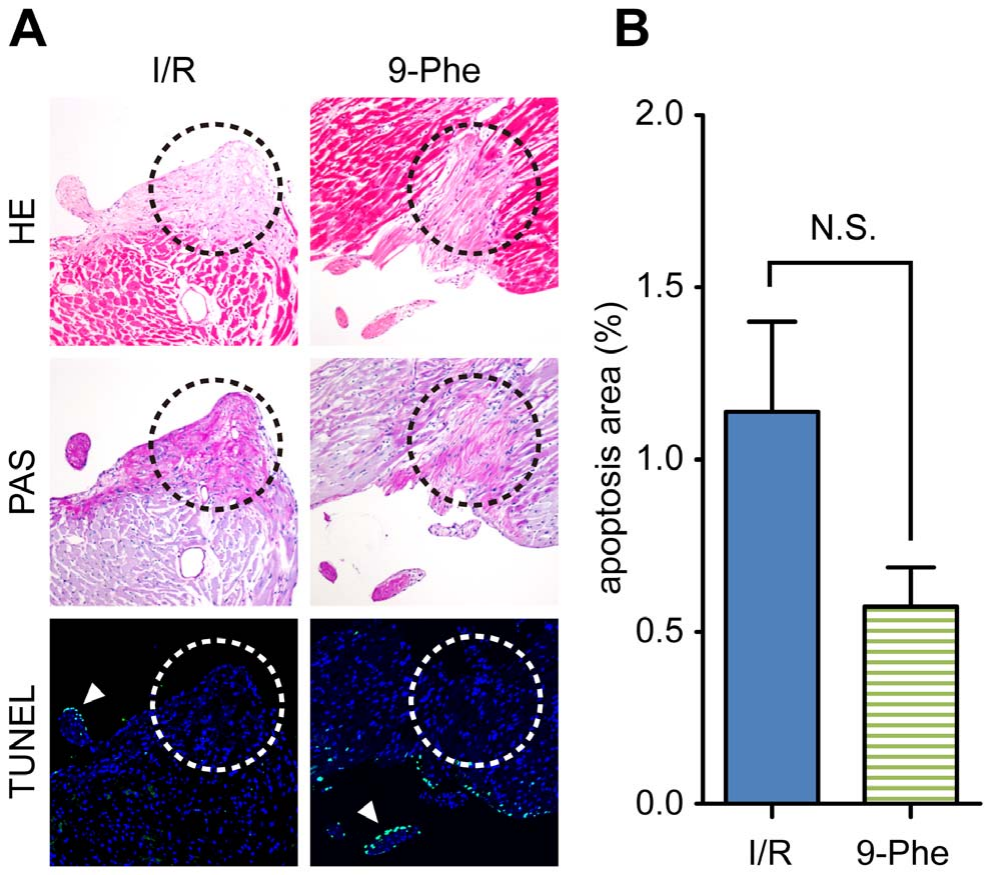
Analysis of cardiac apoptosis. **A.** Detection of apoptosis using the TUNEL assay. Representative microscopic pictures of fluorescein labeled apoptotic cells (green) and counter staining with DAPI (blue) from the I/R and the 9-Phe-treated groups, along with serial sections stained by HE and PAS. Circles show the cells of the cardiac conduction system, which are lightly stained in HE staining and purple in PAS staining. Apoptotic cells were not found in these areas either in the I/R group or in 9-Phe-treated group. Arrowhead: apoptotic cells in the endocardium. **B.** Comparison of apoptosis area between the I/R and 9-Phe-treated hearts. Percentage of apoptotic cells was determined for the whole area of the paraffin section and were expressed as a percentage of the DAPI-positive area. N.S.: *p*>0.05.

To assess the extent of cellular apoptosis within the heart, TUNEL assay was performed (Figure 8). TUNEL positive cells were observed at the endocardium, cardiomyocyte, and vascular cells. However, there seems to be no distinct difference in the expression pattern between the I/R and 9-Phe-treated groups. Although apoptotic cells were observed at the cells surrounding PAS-stained cells in some cases, there was no overlap between PAS-stained cells and TUNEL-positive cells in either the I/R or 9-Phe groups. Regarding the apoptotic cells in the whole areas of the heart sections, although the apoptosis area in the 9-Phe-treated group (0.57±0.11%, n = 6) was smaller than that in the I/R group (1.13±0.26%, n = 6), the difference was not statistically significant (*p*>0.05, Student’s t-test).

## Discussion

### The Cardioprotective Effect of 9-phenanthrol

Here we show that the application of the TRPM4 inhibitor, 9-phenanthrol, to ischemic and reperfused isolated rat hearts reduced myocardial dysfunction as evidenced by the presence of significant functional improvement and reduction in infarct size. These results suggest that 9-Phe protects against myocardial injury during I/R. These results were also supported by *in vivo* experiments using left anterior descending coronary artery occlusion model in a small number of rats (Supplementary Results in File S1: Effect of 9-Phe on I/R injury in anesthetized rats).

### The Role of ATP-sensitive Potassium Channels in the Cardioprotective Effect of 9-phenanthrol

A number of studies have shown that activation of ATP-dependent potassium channels in mitochondria is required for cardioprotection promoted by ischemic preconditioning [3], [21]. In the present study, an inhibitor of ATP-dependent potassium channels (5-HD) was administered together with 9-Phe before ischemia, which is expected to inhibit the classic cardioprotection pathway. However, the LVDP, heart rate, LDH, and TTC data show that the inhibitory effect of 5-HD was not significant. When added alone, 5-HD has no effect on hemodynamic parameters and infarct size in rat models of I/R injury [15], [22]. Our results suggests that the cardioprotective effect of 9-Phe on ischemic–reperfusion injury is not mainly derived from the K_ATP_ pathway. Meanwhile, it is interesting to note that the contractility of the heart in the 5-HD group at 30 min after reperfusion was similar to that in the control group and significantly lower than that in the 9-Phe-treated group (Figure 3). This implies that the cardioprotective effect observed in this study is composed of multiple parts, and at least a part of it is through the K_ATP_ pathway. It is important to note that classic preconditioning is less effective for hypertension [23] and diabetes [24], which predispose to the development of ischemic heart disease. Our findings presented here indicate that treatment with 9-phenanthrol may impart cardioprotective effects upon patients with hypertension and diabetes. We believe that this is a worthy topic for further research.

### Possible Mechanism Underlying the Cardioprotective Effect of 9-phenanthrol

TRPM4 has been linked to diverse physiological functions such as protection against Ca^2+^ overload, regulating the levels of intracellular ATP and reactive oxygen species, and cell death [25], [26], [27], [28]. Recently, Schattling et al. reported that TRPM4 in neurons contributes toward inflammation-induced neurodegeneration by mediating cell death [29], which corresponds to the cardioprotective effect of 9-phenanthrol detected here. There is a possibility that the damage induced by I/R was caused by TPRM4-dependent cell death and that 9-phenanthorol induces cardioprotection by blocking this pathway. To assess whether 9-phenanthrol has a direct action on cardiomyocytes, we employed the H9c2 cardiomyocyte cell line and administered H_2_O_2_ that mimics the condition under ischemia. However, when we applied 9-phenanthrol to the H9c2 cells before treating them with H_2_O_2_, no significant protective effect was observed (Supplementary Results in File S1: Effect of 9-Phe on H9c2 cells exposed to H_2_O_2_), although TRPM4 channels are also expressed in this cell line (Supplementary Results in File S1: TRPM4 gene expression in H9c2 cardiomyocytes). This indicates that 9-phenanthrol may not exert a direct cardioprotective effect on cardiomyocytes.

In the present study, the 9-Phe-treated group showed anti-arrhythmic effect on the I/R injury (Figure 4). Although the anti-arrhythmic effect of 9-Phe in mouse ventricle underwent hypoxia and re-oxygenation was already reported by Simard et al [13], the underlining mechanism seems to be different, as the direct effect of 9-Phe diminishes several minutes after wash out [12]. In the present study, 9-Phe was washed out before 30 min ischemic procedure. Thus, the cardioprotective effect of 9-Phe is probably derived from subcellular processes subsequent to direct activation of TRPM4 channels. The level of expression of transcripts encoding TRPM4 indicates that these channels are expressed to a greater extent in Purkinje fibers than in the septum, atrium, and ventricles [10]. Hence 9-Phe may induce cardioprotection mainly by protecting the conduction system of the heart but not through the cardiomyocytes. In the present study, cells with rich-glycogen, which are characteristic of the cardiac conduction system, seemed not apoptotic in the I/R group, even though the surrounding cells showed severe damage such as swelling or apoptosis. According to Sayk et al [30], the damage of the conduction system after ischemia–reperfusion injury is due to necrosis, not apoptosis. Further studies are required to elucidate the 9-phenanthrol effect on the cardiac conduction system.

### Conclusions

In this study, we showed that 9-phenanthrol induces cardioprotection of isolated rat hearts, most likely due to its inhibitory effect on TRPM4 channels. Although further research, including more detailed *in vivo* experiments than those in this study is required, our findings suggest that TRPM4 may serve as an effective pharmacological target for cardioprotective treatment strategies.

## Supporting Information

Figure S1 **Confirmation of successful LAD occlusion.** Representative mid-myocardial cross sections of evans blue perfused heart. The blue-staining areas represent non-ischemic zone.
(TIF)

Figure S2 **Response of H9c2 cardiomyocytes to oxidative stress.** Cell viability was measured using an MTT assay. Approximately 40% of H9c2 cells survived the 30-min treatment with H_2_O_2_. There were no significant differences between the DMSO and 9-Phe-treated groups (n = 3 for each group).
(TIF)

Figure S3 **TRPM4 gene expression in H9c2 cardiomyocytes.** Expression of TRPM4 mRNA using RT-PCR. Gene expression was confirmed in H9c2 cardiomyocytes, the rat heart, and brain. Samples prepared in the absence of reverse transcriptase served as the negative control.
(TIF)

File S1 **Supplementary methods and results.**
(DOCX)
