# Supplementary figures and images for: 9-Phenanthrol, a TRPM4 Inhibitor, Protects Isolated Rat Hearts from Ischemia–Reperfusion Injury
Source: PLoS One. 2013 Jul 25;8(7):e70587. doi: 10.1371/journal.pone.0070587 (PMC3723883; doi:10.1371/journal.pone.0070587)

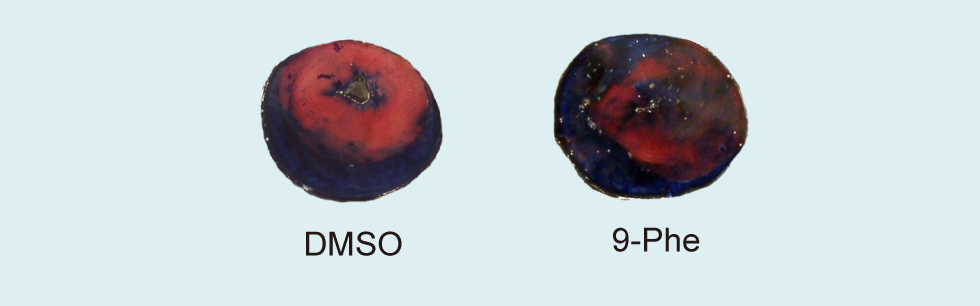

Supplement: Figure S1 — Confirmation of successful LAD occlusion. Representative mid-myocardial cross sections of evans blue perfused heart. The blue-staining areas represent non-ischemic zone. (TIF) [file pone.0070587.s001.tif]

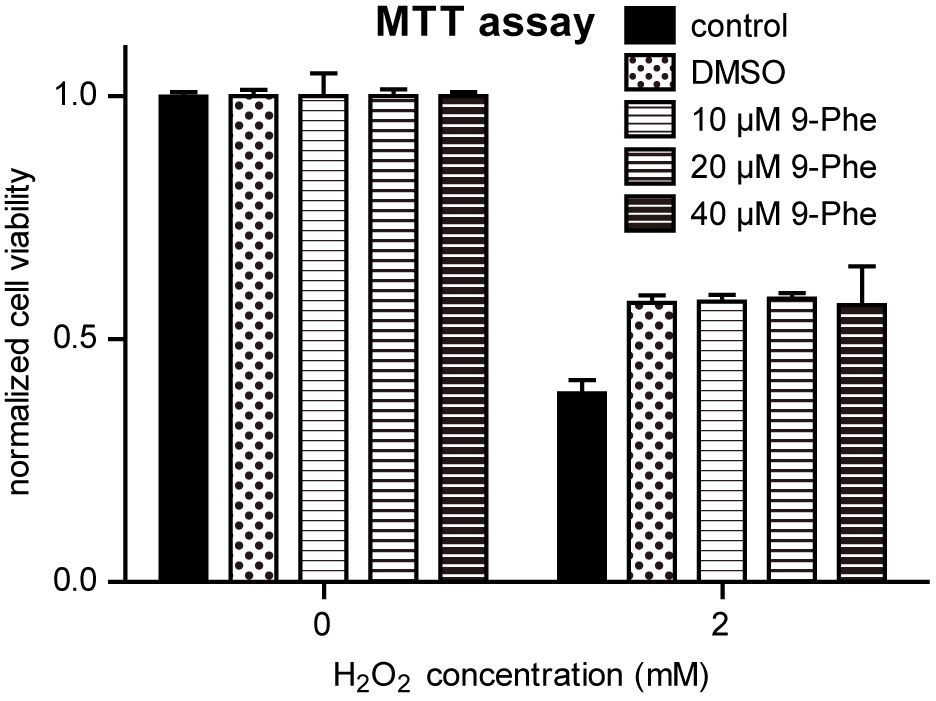

Supplement: Figure S2 — Response of H9c2 cardiomyocytes to oxidative stress. Cell viability was measured using an MTT assay. Approximately 40% of H9c2 cells survived the 30-min treatment with H2O2. There were no significant differences between the DMSO and 9-Phe-treated groups (n = 3 for each group). (TIF) [file pone.0070587.s002.tif]

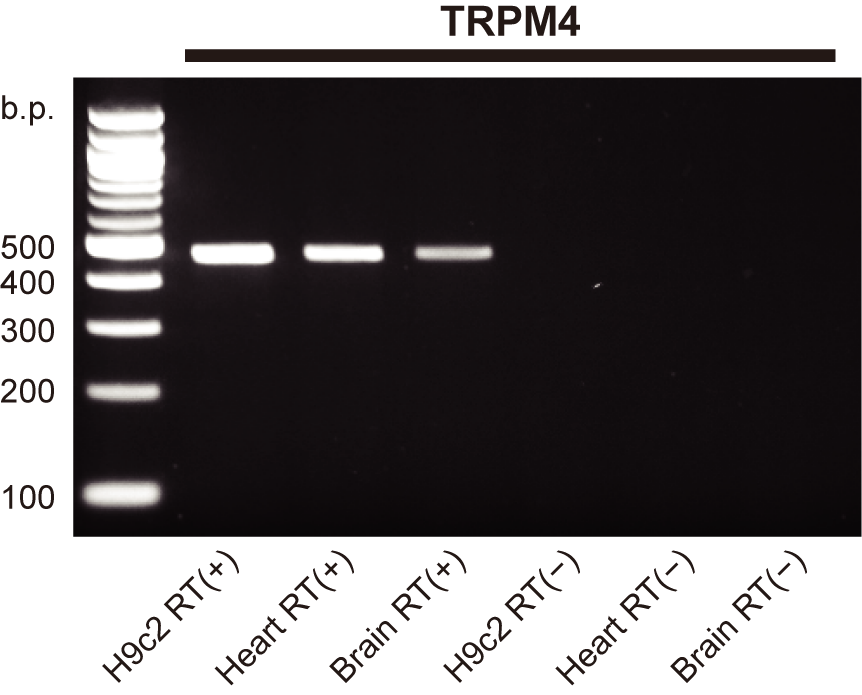

Supplement: Figure S3 — TRPM4 gene expression in H9c2 cardiomyocytes. Expression of TRPM4 mRNA using RT-PCR. Gene expression was confirmed in H9c2 cardiomyocytes, the rat heart, and brain. Samples prepared in the absence of reverse transcriptase served as the negative control. (TIF) [file pone.0070587.s003.tif]
