# Supplementary material for: 9-Phenanthrol, a TRPM4 Inhibitor, Protects Isolated Rat Hearts from Ischemia–Reperfusion Injury
Source: PLoS One. 2013 Jul 25;8(7):e70587. doi: 10.1371/journal.pone.0070587 (PMC3723883; doi:10.1371/journal.pone.0070587)
Supplement: File S1 — Supplementary methods and results. (DOCX) [file pone.0070587.s004.docx]

**Supplementary Methods**

*Assessment of the cardioprotective effect of 9-Phe on I/R injury* in vivo

Anesthesia was induced by intraperitoneal injection of 50 mg/kg pentobarbital. Surgery was performed under artificial ventilation using a small animal ventilator (SAR-830/P, CWE, USA). Body temperature was maintained at approximately 37.5°C with a heating pad. The cardiac function was monitored with a lead II electrocardiogram. Left thoracotomy was performed at the fourth intercostal space, and the left anterior descending coronary artery (LAD) was occluded with a 7-0 prolene suture. A bolus injection of 0.5 ml 9-Phe (10 mg/kg of the rat body weight) or vehicle (DMSO) was administered via the jugular vein 15 min before the occlusion procedure. The rats were subjected to a 30-min period of ischemia by ligating LAD, which was followed by a 90-min period of reperfusion by loosening the ligature. Thereafter, the heart was rapidly removed to check the occlusion procedure. LAD was re-occluded, and the heart was infused with 2 ml of 5% Evans blue via the aorta in order to distinguish between ischemic and nonischemic areas.

*Assessment of the ability of 9-Phe to protect cardiomyocytes from the effects of hydrogen peroxide*

H9c2 cells were obtained from American Type Cell Culture (ATCC, Manassas, VA, USA). The cells were maintained in Dulbecco’s modified essential medium with 10% FBS at 37°C in an atmosphere of 5% CO_2_. The cells were plated in 96-well plates at a density of 2 × 10^4^ cells/100 μL per well and treated with 9-Phe or DMSO 24 h after plating. Thirty wells were randomly divided into 5 groups, and the cells were pretreated with culture medium, DMSO, or 9-Phe (10, 20, or 40 μM). They were then incubated with 2 mM H_2_O_2_ or medium for 30 min. Cell viability was measured using the MTT [1] Cell Proliferation Assay Kit (Cayman Chemical Company, Ann Arbor, MI, USA) according to the manufacturer’s instructions. Viability was determined using a microplate reader at an absorbance of 570 nm.

*Detection of TRPM4 mRNA in H9c2 cardiomyocytes using RT-PCR*

Total RNA of H9c2 cells was isolated using the RNAqueous 4PCR Kit (Life Technologies) according to the manufacturer’s instructions. For total RNA samples of rat heart and brain, FirstChoice Rat Total RNA (Applied Biosystems) was used. Total RNA (1 μg) from each sample was reverse transcribed to cDNA with random hexamer primers using Verso cDNA Kit (Thermo Scientific). PCR primers specific to the TRPM4 channels were used according to Yang et al [2]. Sense primer: 5’-GAGAGGATCATGACCCGAAA-3’, Antisense primer: 5’-GAACTTGCCCCACATTAGGA-3’. Expected PCR product size is 443 base pairs. PCR reactions were performed using AmpliTaq Gold DNA Polymerase (Applied Biosystems) with the following parameters: denaturation at 94°C for 30 s, annealing at 56°C for 45 s, and extension at 72°C for 90 s. A total of 35 cycles was performed, followed by a final extension at 72°C for 10 min. To ascertain that there was no genomic DNA contamination, parallel reactions were run for each RNA sample in the absence of reverse transcriptase. The PCR products were analyzed on 1.8% agarose gel.

**Supplementary Results**

*Effect of 9-Phe on I/R injury in anesthetized rats*

To test whether 9-Phe shows cardioprotective effects on I/R injury in live animals, *in vivo* experiments were performed in rats. After bolus injection of 10 mg/kg 9-Phe, a moderate decrease in heart rate was observed. In the DMSO-injected group, 3 of 5 rats died within 30 min of the ischemia–reperfusion procedure. In contrast, all 3 rats in the 9-Phe -injected group survived the 90 min reperfusion period. Evans blue staining confirmed that a portion of the left ventricle was not stained with the dye in all the experiments, illustrating that the LAD occlusion procedure was successful in all cases (Figure S1).

*Effect of 9-Phe on H9c2 cells exposed to H_2_O_2_*

Postischemic myocardial infarction is mediated by the generation of reactive oxygen species, such as those generated by H_2_O_2_ [3]. To test whether 9-Phe exerts a cardioprotective effect against I/R injury of cardiomyocytes, H_2_O_2_ was applied to H9c2 cardiomyocytes and cell viability was assessed. Figure S2 shows that approximately 40% of the H9c2 cells survived (normalized to the values of untreated cells) the 30-min H_2_O_2_ treatment. Of the groups treated with H_2_O_2_, the normalized cell viability was 0.39 ± 0.03 in the I/R group (n = 3) and 0.57 ± 0.02 in the DMSO group (n = 3), respectively. There was no difference in cell viability between the DMSO and 9-Phe groups [0.58 ± 0.02, 0.58 ± 0.01, and 0.57 ± 0.10 at 9-Phe concentrations from 10 to 40 μM (n = 3 for each concentration)].

*TRPM4 gene expression in H9c2 cardiomyocytes*

To evaluate whether TRPM4 is present in the H9c2 cardiomyocytes, we performed RT-PCR analysis. The rat heart and brain were used as positive controls. As can be seen in Figure S3, TRPM4 gene (443 bp RT-PCR product) was expressed in H9c2 cells, the rat heart and brain. The PCR product was not observed in the samples without reverse transcription.

**References**

1. Sanbe A, Daicho T, Mizutani R, Endo T, Miyauchi N, et al. (2009) Protective effect of geranylgeranylacetone via enhancement of HSPB8 induction in desmin-related cardiomyopathy. PLoS One 4: e5351.

2. Yang XR, Lin MJ, McIntosh LS, Sham JS (2006) Functional expression of transient receptor potential melastatin- and vanilloid-related channels in pulmonary arterial and aortic smooth muscle. Am J Physiol Lung Cell Mol Physiol 290: L1267-L1276.

3. Bolli R (1988) Oxygen-derived free radicals and postischemic myocardial dysfunction ("stunned myocardium"). J Am Coll Cardiol 12: 239-249.
